# Ultrasound Elastography in the Assessment of the Intestinal Changes in Inflammatory Bowel Disease—Systematic Review

**DOI:** 10.3390/jcm10184044

**Published:** 2021-09-07

**Authors:** Dominika Ślósarz, Elżbieta Poniewierka, Katarzyna Neubauer, Radosław Kempiński

**Affiliations:** Department of Gastroenterology and Hepatology, Wroclaw Medical University, Borowska 213, 50-556 Wroclaw, Poland; dominika.slosarz@student.umed.wroc.pl (D.Ś.); elzbieta.poniewierka@umed.wroc.pl (E.P.)

**Keywords:** ultrasound elastography, strain elastography, acoustic radiation force impulse, shear wave elastography, inflammatory bowel disease, ulcerative colitis, Crohn’s disease, fibrosis

## Abstract

Inflammatory bowel disease (IBD) is a chronic condition affecting primarily the gastrointestinal tract and characterized by growing incidence worldwide. Complex diagnostic process of IBD as well as evaluation of disease activity and intestinal complications that are crucial for the therapeutic decisions, require repetitive, invasive, expensive, time-consuming and poorly tolerated tests. In contrast to endoscopy and computed tomography, ultrasound elastography (UE) is non-invasive, non-radiating and non-contrasting dependent tool which might be utilized in IBD patients for the assessment of the intestinal changes. Therefore, we performed the systematic review to evaluate the possible application of the ultrasound elastography for assessment of the intestinal changes in IBD. After the search of three databases: PubMed, World of Knowledge and Scopus, we identified 12 papers which were included in the final analysis. The majority of the studies were focused on the evaluation of the symptomatic ileal/ileocolonic strictures in Crohn’s disease patients that required surgical resection. Only one study concerned ulcerative colitis. The authors evaluated different UE techniques: strain elastography (SE), acoustic radiation force impulse (ARFI) and shear wave elastography (SWE). Results were expressed with semi-quantitative color mapping and strain measurement. Histological scores of inflammation and fibrosis in Crohn’s disease were used as a reference test in the majority of studies. Ultrasound elastography seems to be a promising novel imaging technique supporting evaluation of the intestinal strictures in Crohn’s disease patients in respect to fibrosis detection as well as differentiation between fibrosis and inflammation. However, further research is needed to establish the position of ultrasound elastography in IBD management.

## 1. Introduction

Inflammatory bowel diseases (IBD) are chronic, relapsing and remitting conditions affecting primarily the gastrointestinal tract characterized by rising incidence worldwide [1]. The major types of IBD are ulcerative colitis (UC) and Crohn’s disease (CD). UC is a chronic mucosal and submucosal inflammation limited to the colon and rectum, whereas in CD, a whole intestinal wall is inflamed and every part of the gastrointestinal tract may be involved [2]. Still, both conditions share similar and not fully elucidated pathogenesis including interplay between genetic, microbiological and environmental factors. According to the recently published study, genetic vulnerabilities in IBD are concerning pathways regulating homeostasis between the immune system, mucosal barrier tissues and the microbiome [3]. As a consequence of complex pathogenesis, single diagnostic test for IBD is still missing. The diagnosis is established based on the combination of symptoms, endoscopic tests and imaging examinations (computed tomography, (CT) and/or magnetic resonance (MR)) together with histopathological assessment. Invasive, time-consuming and expensive endoscopy with biopsy sampling for histopathology evaluation remains a crucial diagnostic tool in IBD [4]. Further, as clinical symptoms poorly correlate with intestinal disease activity, this last one is evaluated by a set of laboratory indices (C-reactive protein (CRP), fecal calprotectin), endoscopy and imaging tests. However, CT should be reserved for emergency cases due to high radiation exposure [5]. Furthermore, assessment of the effectiveness of treatment strategy requires endoscopy as other indices of mucosal healing (MH) are still unavailable [6].

In recent years, there has been growing interest in the utility of ultrasound elastography (UE) as a novel tool to evaluate tissue stiffness [7]. Over time, literature has elaborated on elastography implications in assessment of liver, breast, thyroid, kidney, prostate, lymph nodes and pancreas [8,9,10,11]. Elastography techniques might be divided into qualitative and quantitative methods [12,13]. Strain elastography (SE) is a basic qualitative elastography technique. Quantitative methods are used in transient elastography (TE), point shear wave elastography (pSWE), also known as acoustic radiation force impulse (ARFI) and shear wave elastography (SWE). SE is based on palpation with transducer or physiological patient movement to generate pressure on tissues and their displacement, which are decreased in cases of stiffer structure. The use of both ultrasound imaging and elastography lead to tissue stiffness color mapping [12]. In turn, TE uses an ultrasound probe and transducer supplying low frequency (50 Hz) vibration to induce underlying tissues by a shear wave. The probe has a pulse-echo ultrasound to receive and measure the propagation of shear waves and their velocity which correspond to tissue stiffness [14]. In ARFI imaging, there are generated, localized, impulsive acoustic radiation forces conducting a dynamic tissue displacement and subsequently, tissue recovery to its original position. Inducted sheared waves in combination with ultrasound imaging ARFI facilitate the assessment of tissue stiffness in a selected tissue region of interest (ROI) [15]. An apparent limitation of the ARFI imaging is preset, constant size of ROI, and no information about standard deviation. SWE is a quantitative method based on an ultrasound probe generating radiation force and inducing simultaneously shear waves from different focal points. It influences the creation of the conical shear wave front. Progression of shear waves activates ultrasound images which are successively compared. As an outcome of induced tissue displacement, ultrasound imaging and its comparative peculiar image of tissue stiffness are being generated. The real-time image and capacity to measure tissue stiffness in a chosen direct area are the advantages of SWE [16].

Our goal was to provide an overview of ultrasound elastography as a potential diagnostic tool in inflammatory bowel disease to address the question whether it can support the stratification of IBD patients. Therefore, we conducted a systematic review of literature in order to investigate the utility of ultrasound elastography in assessment of intestinal changes in IBD.

## 2. Materials and Methods

**Figure 1:**
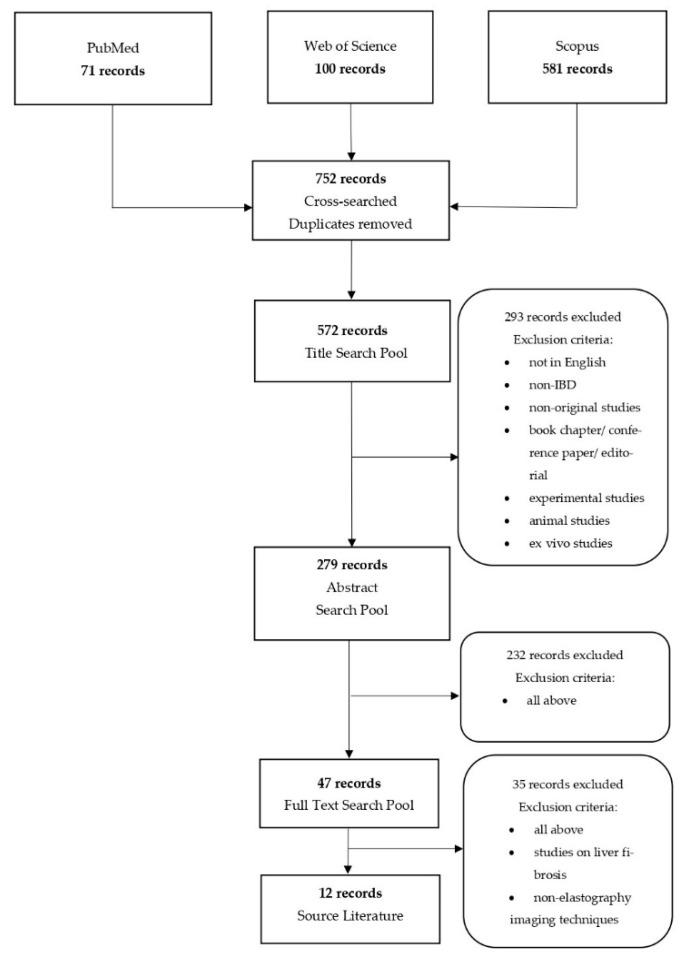
Flowchart presenting the selection process.

To review the role of elastography in IBD patients, we have searched three publication databases: PubMed, World of Knowledge and Scopus. Combinations of the following keywords were used: (“elastography” or “ultrasound” or “SE” or “shear wave elastography” or “SWE” or “acoustic radiation force impulse” or “ARFI”) AND (“Crohn’s disease” or “ulcerative colitis” or “inflammatory bowel disease” or “IBD”). The search was limited to publications published between January 2015 and June 2021. Analysis of data was conducted according to PRISMA recommendations. Duplicate records from the databases were removed prior to the first eligibility screening. Exclusion criteria were as follows: experimental studies (including animal studies and in vitro research), non-IBD, non-original articles, on liver elastography in IBD and non-English language. Finally, we enrolled 12 publications to review. The search strategy has been summarized in Figure 1.

## 3. Results

### 3.1. Interpretative Synthesis of Data: Elastography in CD

A growing body of literature has investigated the applicability of ultrasound intestinal elastography in CD due to the whole wall inflammation and common fibrosis. The preliminary studies on this field were carried out with animal models and human subjects [17,18,19]. Since 2015, much more information on the usability of elastography in CD patients has become available [20,21,22]. Each of them used ultrasound elasticity imaging (UEI), although they implicated different reference methods. Nevertheless, studies suggested that ultrasound elastography may be a feasible technique for assessment of intestinal wall fibrosis in CD patients.

Baumgart et al. conducted research with 10 patients who had been elected for surgical intestinal resection due to the symptomatic stenosis. In order to perform an intestine evaluation, they examined bowel segments affected and unaffected by CD before, during and after surgery. Measurements of the intestinal wall had been performed via a built-in press guide function to generate repetitive compression and by direct tensiometry strain measurements intraoperatively. Tests revealed that the affected bowel segments had higher strain than unaffected. Furthermore, there was a significant correlation between histopathology findings, such as collagen content and elastography as well as tensiometry. The authors concluded that ultrasound-based real-time elastography can be used to reliably (*p* < 0.001) distinguish fibrotic from nonfibrotic tissue [20].

In turn, Fraquelli et al. assessed bowel strictures in 23 CD patients qualified for surgery and 20 CD patients with active non-stricturing/non-penetrating disease. The evaluation was based on a comparative analysis between both the semi-quantitative visual color scale and ultrasound elastography imaging strain ratio measurements, and histopathological findings in respect to fibrosis and inflammation. The most remarkable result was the correlation of strain ratio and fibrosis severity. Study revealed that the UEI strain ratio had an excellent discriminatory ability for diagnosing severe bowel fibrosis, as assessed by AUROC (strain ratio: 0.917; 95% CI, 0.788–1.000; color scale: 0.680; 95% CI, 0.460–0.899). From among standard US parameters, bowel wall thickness was significantly correlated with EI strain ratio. Moreover, patients with non-surgical, inflammatory CD had a lower strain ratio rather than surgical patients (*p* = 0.0005). No significant correlation was observed between color scale, stratification pattern and strain ratio [21].

Furthermore, Fufezan et al. introduced a novel method to evaluate the structure of the intestinal wall. They conducted strain elastography in 14 pediatric CD patients in addition to hydrosonography (HS), clinical data and MRI (in 6 selected cases). They reported the correlation between HS findings, color doppler and SE pattern and strain ratio values (*p* < 0.005). Moreover, they showed correlation between disease activity markers and SE strain ratio value (*p* < 0.005) but not with fecal calprotectin level (*p* = 1065). Additionally, they put forward a color code to evaluate the inflammation and fibrosis of the intestinal wall. The main presumption was based on color code corresponding with tissue stiffness (“red-soft, green-intermediary stiffness, blue-hard”) [22]. Due to elastography image, they distinguished three types of wall pattern: bowel in remission (“blue/green/blue”), inflammatory wall (“green/blue”), fibrotic wall (“blue”; no color stratification). The main weakness of the study is that authors did not correlate color stratification scale to histopathology evaluation.

Further research focused on proposing color patterns to assess intestinal fibrosis. Sconfienza et al. put forward an idea to divide SE axial image of the terminal ileum into eight equal parts. MRI was the reference technique in this experiment. Thereafter, each sector had to be estimated subsequently by a scale in which red = 1 (minimal fibrosis), green = 2 (intermediate fibrosis) and blue = 3 (maximal fibrosis). Patients could receive from 8 to 24 points. A higher score was related to increased fibrotic tissue in the intestine. In the conclusion, the researchers reported that this method could possibly be a tool to estimate fibrosis in CD. However, using MRI as a referenced technique is a major flaw of their experiment [23]. Distinguishing fibrotic bowel segments from the inflamed ones by color pattern in compilation with MRI was also presented by Lo Re et al. [24]. 

A different outcome was presented by Serra et al. They enrolled 26 CD patients with symptomatic stricturing disease. Seven days before surgery, each patient underwent ultrasonography with color-Doppler and strain elastography with strain ratio measurement. Results were correlated with a modified scoring system for inflammatory and fibrostenotic features of CD. In contrast with the previous research, Serra et al. reported that mean strain ratio measured during real-time strain elastography did not correlate with a histopathological score of inflammation (*p* = 0.531), fibrosis (*p* = 0.877) and clinical or biochemical markers: Harvey Bradshaw index (HBI) (*p* = 0.879), CRP (*p* = 0.485), previous ani-TNF therapy (*p* = 0.964). Moreover, the color-Doppler did not evaluate either gut inflammation (*p* = 0.764) or fibrosis (*p* = 0.288) [25]. 

A different point of view for ultrasound intestinal elastography was introduced by Quaia et al. The sensitivity and specificity of the conventional B-mode ultrasound, contrast-enhanced ultrasound (CEUS) and real-time strain elastography in combination were investigated. It was suggested that the correlation of these methods may be a visual, supportive tool in distinguishing fibrotic strictures. The research was limited by the application of mucosal deep biopsy as a referenced standard [26]. 

In line with the elastography technique enhancement, there has been a growing interest in ARFI and SWE imaging. In order to evaluate intestinal fibrosis and inflammation, Lu et al. conducted a study with the shear wave elastography using ARFI and contrast-enhanced ultrasound. They enrolled in the study 95 CD patients with stricturing disease. In 15 cases, surgery and histopathology assessment were performed. SWE mean value was significantly higher in a patient with surgery rather than without it (*p* < 0.01). They found out a moderate correlation between SWE and muscular hypertrophy which was more common in bowel strictures rather than fibrosis (*p* = 0.02). Furthermore, SWE did not correspond to fibrosis in histopathology samples [27]. 

Other researchers evaluated the utility of ARFI retrospectively (77 patients) and prospectively (21 patients). Stomach, (neo)terminal ileum and sigmoid were tested with ARFI. They reached the conclusion that, retrospectively, there is a correlation between ARFI and bowel wall thickness, Limberg score, CRP and HBI. Surprisingly, there was no dependence on elastography prospectively. The experiment was marred by using ultrasound parameters as a reference method and the different size of the analyzed group [28].

Further research was managed by Chen et al. [29]. They compared ultrasound image scored by Limberg classification, SWE and histology [30]. The study group consisted of 42 CD patients (26 with strictures in terminal ileum, 9 in colon). They put forward a novel ultrasound classification method. At first, they estimated mild, moderate and severe fibrosis (*p* = 0.008) cut-off values (14.4 ± 2.1 vs. 17.4 ± 3.8 vs. 23.0 ± 6.3 Kpa) in SWE. The experiment was undermined by the lack of a control group to standardize new cut-off value. However, they pointed out that there was a significant correlation between the SWE mean value and intestinal fibrosis in spite of the inflammation [29]. In addition to SWE enhancements, Ding et al. conducted research to equate this method with TE. A comparative analysis of TE, ARFI and histopathology was performed on 25 patients. Both SE and ARFI imagine were divided into fibrosis stage by two visual color scales. They reported significant difference between mean shear wave velocity in inflammatory stenosis and fibrotic stenosis (*p* < 0.05). As a result, it was reported that only SWE achieved equitable sensitivity and specificity in detecting intestinal fibrosis [31]. It suggests that quantitative elastography methods are superior to qualitative (Table 1). 

### 3.2. Interpretative Synthesis of Data: Elastography in UC

We were able to find one study on ultrasound elastography in ulcerative colitis [32]. Goertz et al. performed ARFI shear wave elastography in 20 UC patients. The authors evaluated ascending, transverse, descending and sigmoid colon in UC patients and healthy volunteers in compilation with ultrasound wall thickness assessment and vascularization scale. The ROI region was targeted into the whole wall (collapsed partially in healthy volunteers and diseased in UC patients). The study has shown that ARFI values were higher in UC patients than in controls (*p* = 0.021), especially in transverse (*p* = 0.045) and sigmoid colon (*p* = 0.032). No correlation between ARFI values and wall thickness, Limberg score or clinical Mayo-Subscore raised a concern that the data was not compared to a more validated method, such as MRI or colonoscopy (Table 2).

## 4. Discussion

Inflammatory bowel disease, due to the increasing incidence worldwide, changing age profile of patients, unclear pathogenesis, unavailable simple diagnostic algorithm and missing effective therapy, comprises a currently significant health burden. IBD management is inseparably connected with several imaging modalities which support the diagnosis establishment, stratification of patients and therapeutic decisions. Ultrasound elastography is a novel radiation- and contrast-free option which is assessed in IBD.

As expected, the majority of the studies on UE in IBD were focused on Crohn’s disease patients. From among 11 studies, 5 included patients with symptomatic strictures in ileocolonic region. The authors were able to demonstrate the effectiveness of UE in recognizing fibrotic type of strictures. 

Occurrence of strictures in Crohn’s disease may be associated with inflammation, fibrosis or malignancy. Fibrosis in CD refers to accumulation of collagen and fibronectin extracellular matrix (ECM) produced by mesenchymal cells, such as fibroblasts, myofibroblasts and smooth muscle cells as an effect of chronic inflammation and increased levels of cytokines, chemokines and growth factors [33,34]. Previous research has shown that 4.6% of the patients had stricturing disease and the cumulative risk of developing com-plication increases to 50.8% after 20 years of the disease duration [35]. As recently reviewed by Mak and Ng, up to 20% of CD patients present with strictures at diagnosis that may arise, similarly to the inflammatory changes in every segment of the gastrointestinal tract affecting, the most frequently, ileum alone (30–45%) and ileocolonic region (40–60%). Moreover, despite being found less often than in CD patients, strictures are also found in 1–11% of UC patients [36]. Despite the huge progress in the implementation of novel therapeutic agents into clinical practice, it was demonstrated that they do not influence the occurrence of fibrosis. In a recently published systematic review on nonsurgical therapy of CD-related strictures from among drug therapy only, anti-TNFα agents appeared to be effective in preventing surgery as during 4 years of follow-up, 50% of patients could escape operation [37]. Unfortunately, antifibrotic therapy is not available. Several options of endoscopic and surgical interventions for strictures related with CD have been developed in order to resolve clinical symptoms and improve a patient’s quality of life. Therapeutic approach varies up from stricture pathology, which may be fibrotic, inflammatory, mixed or, the most importantly, malignant. Therefore, precise preoperative evaluation of the stenosis character has a crucial role for the therapeutic strategy [38]. 

Recognizing the intestinal complications of IBD involves endoscopy as well as CT or MR imaging. MRI or/and intestinal ultrasound are recommended in the detection of strictures in CD. There is no imaging technique to evaluate the degree of fibrosis [39]. Further, biomarkers of fibrostenosing Crohn’s disease are unavailable, despite some such as cartilage oligomeric matrix protein, hepatocyte growth factor activator, and lower levels of microRNA-19-3p which are promising [40]. Several options have been proposed to assess fibrosis in CD with some focusing on MRI, others on ultrasound elastography [41,42]. 

Yet, due to the heterogenicity of the studies included in our analysis, drawing out the final conclusions has to be done carefully. First of all, the authors applied various ultrasound elastography techniques which results from the constant development of this diagnostic modality. For instance, as described above, every UE option has a distinct way to provide findings, from semi-quantitative color maps to quantitative results. Yet, there are different cut-off values proposed for diagnosing fibrosis. Secondly, the authors employed various reference standards: histology, MRI, hydrosonography and ultrasonography. Moreover, among the studies with histological reference, there were different histological scores utilized. In line with development of research on ultrasound elastography, the reference techniques to distinguish intestinal inflammation and fibrosis are needed. Currently, there is no gold standard in intestinal fibrosis assessment, however, histological evaluation is claimed to be the most aware. Still, there is no general agreement on histology scoring of fibromuscular stenosis [30]. Second, the most frequently applied method after histology was MRI. In recent years, there has been a growing interest in MRI feasibility in detecting and evaluating intestinal fibrosis. Various approaches have been proposed to solve the issue. Diffusion-weighted MRI (DW-MRI), contrast-enhanced MRI (CE-MRI) and magnetization transfer MRI (MT-MRI) are suggested to distinguish fibrosis and inflammation. However, there is no validated imaging technique to assess the degree of fibrosis [41].

Prior types of research were focused on SE. However, there is an increasing number of studies on ARFI and SWE imaging. Early studies tended to be focused on color fibrosis mapping and later, on strain measurement. Previously published articles have a few major flaws. Firstly, all of them were conducted in small groups. Secondly, approximately only half of the introduced studies used histology as a reference method. Histology claims to be the most relative technique to estimate intestinal fibrosis, in spite of the fact that there is still no general agreement on the histological scoring system of stenosis in CD [30]. In other types of research, elastography was compared to the less validated methods, such as MRI, hydrosonography, mucosal deep biopsy, ultrasound parameters (bowel wall thickness and intramural semi-quantitative vascularization grade) and/or clinical data. 

In spite of the fact that UC affects rectal or/and colonic mucosa, so the elastography may not be as effective as in CD, several studies were conducted to assess elastography implication in UC. One of the first researches on elastography in ulcerative colitis was conducted by Ishikawa et al. in a group of 37 patients with left-sided colitis or pancolitis [43]. Authors applied real-time tissue sonoelastography (EG), a method which assesses tissue stiffness during ultrasound by freehand compression. In turn, Rustemovic et al. investigated the applicability of transrectal ultrasound (TRUS) elastography in IBD patients. The authors concluded that transrectal ultrasound elastography is a promising diagnostic tool in distinguishing CD and UC [44]. After January 2015, we were able to find only one study which was focused on elastography in UC. The authors demonstrated that ARFI values in UC patients were higher than in controls. However, the study was conducted on a small group of patients and the reference method was also debatable.

Limitations of our research result from the heterogenous character of available studies on ultrasound elastography of intestines in IBD. First of all, included studies applied different techniques of ultrasound elastography and various reference standards. Furthermore, this novel imaging method is continuously developing.

## 5. Conclusions

In conclusion, elastography is a promising novel imaging technique to evaluate intestinal strictures in Crohn’s patients. Further research on larger groups of patients with both CD and UC are needed in order to establish the ultrasound elastography protocol and to validate cut-off values of intestinal wall fibrosis. 

## Figures and Tables

**Table 1:** Ultrasound elastography of intestines in Crohn’s disease.

Author	Year	Total Subjects Included	Ultrasound Elastography Technique	Reference Standard	Main Findings
Baumgart et al. [20]	2015	10 CD patients with ileocolonic CD and symptomatic stenosis that required surgery	Pre-, intra- and post-operative RTE	Histologic evaluation (morphometrics, disease activity, fibrosis); direct tensiometry strain measurement	The aggregated RTE strain mean values were significantly higher in unaffected than in affected gut segments (mean, 169.0 ± 27.9 vs. 43.0 ± 25.9; *p* < 0.001). There was significant association between RTE and collagen depositions.
Fraquelli et al. [21]	2015	23 CD patients that required surgery; 20 CD patients with active non-structuring and non-penetrating disease	UEI of terminal ileum by means of color scale and quantitative strain ratio measurement	Semi-quantitative and quantitative histological image analysis: scores for fibrosis (mild/moderate versus severe) and acute/chronic inflammation (AIS, CIS)	The ileal strain ratio of inflammatory CD patients was significantly lower than in operated CD patients with severe fibrosis and was significantly correlated with the severity of bowel fibrosis at histological analysis; it was characterized by an excellent discriminatory ability for severe bowel fibrosis (AUC: 0.917).
Fufezan et al. [22]	2015	48 bowel segments (30 ileum and 18 colon) in 14 pediatric CD patients	SE	Hydrosonography, clinical data, MRI (6 patients)	SE and SR correlated with disease activity markers (ESR, CRP) and hydrosonography findings.
Sconfienza et al. [23]	2016	16	SE of terminal ileum by means of color map and semi-quantitative scale	MRI enterography	RTS of the terminal ileum in CD may differentiate between fibrotic and inflammatory strictures.
Lu et al. [27]	2017	95 patients; 15 patients had ileal resection	SWE	Histology (scores for inflammation, fibrosis, and muscular hypertrophy); CEUS	SWE mean value was significantly higher in a patient with surgery rather than without it (*p* < 0.01). There was a moderate correlation between SWE and muscular hypertrophy and no association between SWE and fibrosis score.
Serra et al. [25]	2017	26 patients with symptomatic stricturing ileocolonic CD that required resection (29 bowel segments)	SE	Histopathology evaluation of CD (scoring system for inflammatory and fibrostenotic features)	No significant correlation was found between mean strain ratio and fibrosis score (*p* = 0.877).
Lo Re et al. [24]	2017	35 (41 affected bowel segments and 35 unaffected)	SE	MRI	There was a correlation between US-SE color scale and T2 signal intensity, and between the US-SE color scale and ADC maps.
Quaia et al. [26]	2018	20	SE	Mucosal deep biopsy	Combination of the conventional B-mode ultrasound, CEUS and RTSE may support distinguishing of fibrotic strictures.
Goertz et al. [28]	2018	77 retrospectively 21 prospectively	ARFI	Ultrasound parameters (bowel wall thickness and intramural semi-quantitative vascularization grade)	Retrospectively, the ARFI values correlated with the bowel wall thickness and Limberg vascularization score. Prospectively, there was no correlation between ARFI and bowel wall thickness, Limberg score, clinical activity, and CRP. A cut-off analysis of 105 ileal ARFI measurements showed a cut-off value of 1.92 m/s for the diagnosis of ileal inflammation with 75.3% sensitivity and 87.5% specificity.
Chen et al. [29]	2018	35 with ileal/ileocolonic symptomatic strictures that required surgical resection	SWE	Histology (score for fibrotic and inflammatory CD)	The mean SWE value of stenotic bowel wall was significantly higher in severe fibrosis (23.0 ± 6.3 Kpa) than that in moderate (17.4 ± 3.8 Kpa) and mild fibrosis (14.4 ± 2.1 Kpa) (*p* = 0.008). Using 22.55 KPa as the cut-off value in discriminating between mild/moderate and severe fibrosis, the sensitivity and specificity was 69.6% and 91.7% (AUC 0.822, *p* = 0.002).
Ding et al. [31]	2019	25	SE, ARFI, p-SWE	histology	For SE, the optimal cut-off value was a score of 4 or greater (sensitivity, 75%; specificity, 66.7%; accuracy, 68%; PPV, 30%; NPV, 93.3%; AUROC, 0.708; however, *p* > 0.05). For ARFI, the optimal cut-off value was a score of 4 or greater (sensitivity, 50%; specificity, 81%; accuracy, 76%; PPV, 33.3%; NPV, 89.4%; AUROC, 0.669; *p* < 0.05). For p-SWE, the optimal cut-off value was reached when the shear wave velocity exceeded 2.73 m/s (sensitivity, 75%; specificity, 100%; accuracy, 96%; PPV, 100%; NPV, 95.5%; AUROC, 0.833; *p* < 0.05).

ARFI, acoustic radiation force impulse; MRI, magnetic resonance imaging; SE, strain elastography; SWE, shear wave elastography; MR, magnetic resonance; US, ultrasonography; RTE, real-time elastography; AUROC, area under the receiver operating characteristic curve; AIS, acute inflammatory score; CIS, chronic inflammatory score; ADC, apparent diffusion coefficient; CEUS, contrast enhanced ultrasound; CRP, C-reactive protein, p-SWE; point shear wave elastography.

**Table 2:** Ultrasound elastography of intestines in ulcerative colitis.

Author	Year	Total Subjects Included	Ultrasound Elastography Technique	Reference Standard	Main Findings
Goertz et al. [32]	2019	20 UC 13 non-IBD	ARFI	ultrasound	ARFI values were higher in UC than in control group

ARFI, acoustic radiation force impulse; non-IBD, non- inflammatory bowel disease; UC, ulcerative colitis.
